# “Obesity–Years” Burden May Predict Reversibility in Heart Failure With Preserved Ejection Fraction

**DOI:** 10.3389/fcvm.2022.821829

**Published:** 2022-02-07

**Authors:** Anand Chockalingam

**Affiliations:** ^1^Division of Cardiovascular Medicine, Department of Medicine, Five Hospital Drive, University of Missouri, Columbia, MO, United States; ^2^Home Cardiac Rehabilitation Program, The Harry S. Truman Memorial Veterans' Hospital, Columbia, MO, United States

**Keywords:** obesity cardiac remodeling, heart failure, severe obesity, HFpEF–heart failure with preserved ejection fraction, resilience, obesity cardiomyopathy, reversible cardiomyopathy

## Introduction

Obesity is increasing in all age groups and understanding its contribution to Heart Failure (HF) is central for optimizing care. Heart Failure with preserved ejection fraction (HFpEF) is a clinical syndrome where symptoms and signs of HF occur due to high ventricular filling pressure despite normal left ventricular ejection fraction. In recent trials, HFpEF accounts for over half of all heart failure diagnoses (1). Through careful hemodynamic and echocardiographic characterization, “obese HFpEF” has been identified as a distinct phenotype within the larger HFpEF population (2). Compared to “typical HFpEF,” “obese HFpEF” patients have higher BMI and experience onset of clinical decompensation a decade earlier. Recent insights help in better understanding the mechanisms of obesity mediated HFpEF. If recognized early, much of the cardiac pathology of obese HFpEF may be reversible with adequate weight reduction.

## Obesity Trajectory

Childhood obesity in the US (with onset between 2 and 5 years of age) has dramatically increased from 5% in 1970s to 19% in 2018. Severe childhood obesity has similarly increased from 1% in 1970s to 6% in 2018 with corresponding increase in lifetime cardiac workload. HF remains a clinical diagnosis despite recent developments in cardiac imaging and biomarkers. The Framingham criteria were validated in systolic HF patients in the 1960s when prevalence of severe obesity was <1% of the US population. When decompensated HF despite normal systolic function (HFpEF) was first reported in the 1990s, the prevalence of severe obesity was below 10% in the US (3). Earlier HFpEF trials excluded obese patients further limiting our understanding of its real contribution to decompensation. With severe obesity in the US projected to exceed 24% by 2030, reliable tools are needed to quantify cardiac pathology (4).

## Obesity Cardiomyopathy

Obesity cardiomyopathy (OC) describes obesity induced increase in total blood volume and cardiac output with resultant left ventricular dilation, increased left ventricular wall stress, compensatory left ventricular hypertrophy, and left ventricular diastolic dysfunction (5).

Extreme obesity with body mass indexes (BMI) of 40–70 kg/m^2^ can cause OC in younger adults without hypertension, coronary artery disease, and other comorbidities (5). Systolic dysfunction has been variably recognized in association with severe obesity, although the role of confounding etiologies like hypertension, sleep apnea, atrial fibrillation and sepsis cannot be excluded. Similar to tachycardia, peripartum, inflammatory, and Takotsubo cardiomyopathies, OC is also a reversible cardiomyopathy (6). Significant (43 kg) weight loss through gastric bypass in younger adults (30–45 years of age) improves cardiac dimensions, filling pressure and diastolic function, confirming OC is indeed reversible (7). According to the ACC/AHA stages of HF, structural changes of OC would correspond to stage B HF since clinical decompensation is not yet evident in these patients (8).

## “Obesity–Years” Burden

**Figure 1:**
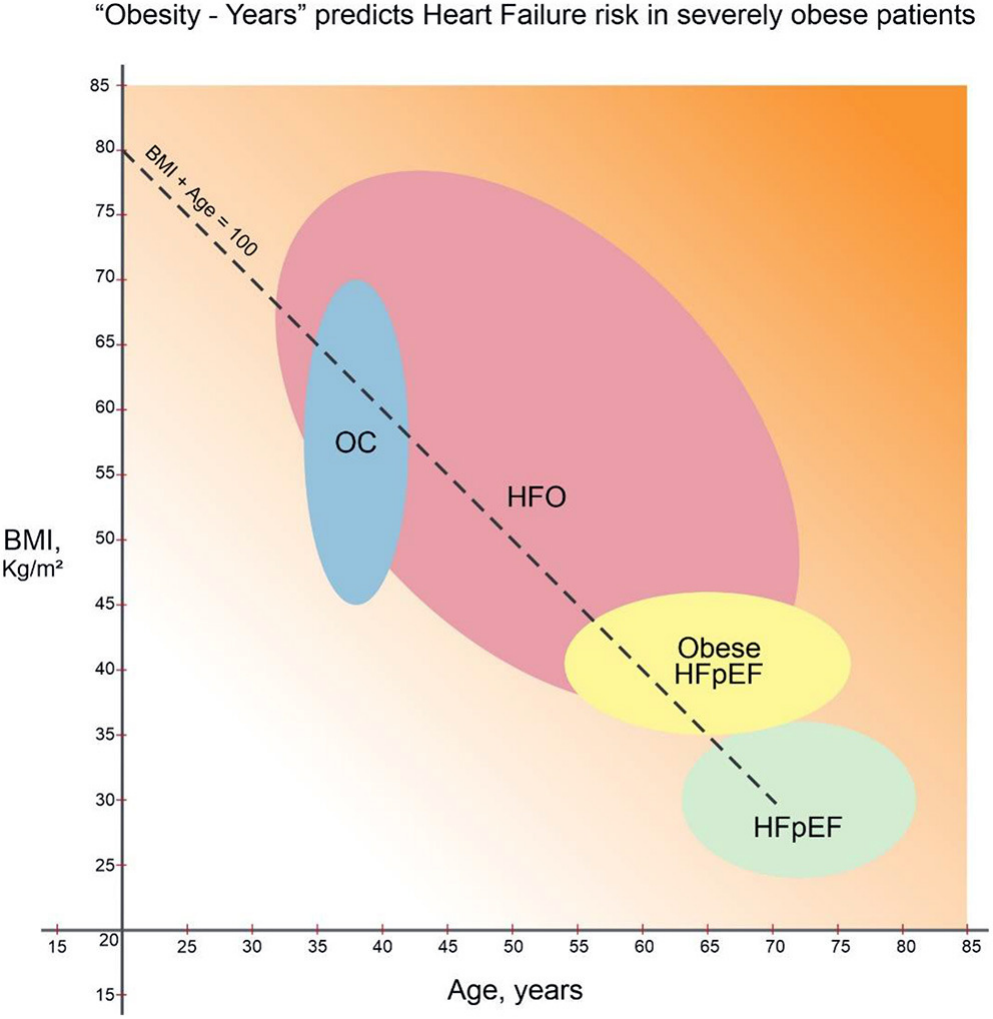
“Obesity–Years” burden and cardiac resilience in heart failure (1, 2, 5, 12). Heart failure with preserved ejection fraction (“typical” HFpEF) (1), obese HFpEF (2), and obesity cardiomyopathy (OC) groups (5) derived from standard deviation ranges for age (in years, X-axis) and BMI (kg/m^2^, Y-axis). Lifetime cumulative risk for HF of obesity (HFO) increases when “obesity-years” (BMI + age) reaches 100. Increasing background gradient denotes declining cardiac resilience with age beyond 70 years. Thus, younger HFO patients can expect resolution of cardiac symptoms and HFpEF reversal with sufficient weight loss (12).

Duration of obesity plays a central role in determining cardiac burden. The probability of OC increased dramatically from 20% when duration of severe obesity was 15 years to 95% at 25 years (9). Patients may not reliably recall their weight throughout life. In busy clinical settings, without periodic visits to the same healthcare system, quantifying obesity burden is challenging. Recognizing the numerous caveats, “Obesity–years” may offer an estimate of lifetime burden of obesity. Cohorts of “typical” HFpEF, obese HFpEF and OC are derived from published age (X axis) and BMI (Y axis) standard deviation ranges in Figure 1 (1, 2, 5). When empirically traced, the intercept of age + BMI = 100 passes through all these 3 cohorts. Thus, in primary care and HF settings, this may represent an arbitrary threshold of HF risk due to obesity. BMI of 40 kg/m^2^ can cause HFpEF by the age of 65 years (2). This hypothetical “obesity–years” threshold (when age + BMI exceeds 100) to predict stage B HFpEF warrants further validation.

## Heart Failure of Obesity

A new term, heart failure of obesity (HFO), implies obesity can account for HF independent of other comorbidities. HFO would include obese HFpEF patients with symptoms and signs of decompensation (stage C) as well as OC patients with structural cardiac abnormalities and diastolic dysfunction (stage B). Weight reduction may improve symptoms even in instances when HF is primarily due to hypertension, ischemic cardiomyopathy, valve disease or renal failure. HFO is likely by the age of 30 years if BMI exceeds 70b kg/m^2^ and when an “asymptomatic” individual with BMI 50 kg/m^2^ reaches the age of 50 years.

Clinical diagnosis of HF is challenging in severe obesity. Clinical and biomarker criteria to diagnose HF have yet to be validated in the severely obese. HFpEF is shifting to increasingly younger populations. We encounter stage C decompensated HF in 30–55-year-old patients when the BMI is 60–80 kg/m^2^. Typical HF symptoms of dyspnea and edema may be attributed to obesity, deconditioning and arthritis. Brain natriuretic peptide increase is blunted in the obese and thus unreliable in diagnosing HFO (10). Often weight precludes catheterization, and echocardiography images are suboptimal. Stage C clinical decompensated HF is widely considered a maladaptive irreversible chronic disease with adverse prognosis (1). In HFO, however, both stages B and C appear to fall along a continuum of compensatory cardiac adaptations to the excess workload. Likelihood of Stage B HFO increases when age + BMI approaches 100. The diagnosis of Stage C HFO requires pulmonary congestion, volume overload or need for diuretics.

## Resilience and Aging

Diverse evidence from aging and resilience research, gastric bypass, and calorie restriction literature suggests potential for reversing OC and obese HFpEF. Broadly, resilience is the ability of a system to maintain specific functions in the face of change. Cardiac resilience denotes the ability to maintain adequate cardiac output despite comorbidities (hypertension, valve disease, or renal dysfunction) and acute stressors (exercise, anemia, surgery, or infection). Resilience declines significantly with aging. Compared to a 30-year-old, the risk of all cause death increases 8-fold by age 56 and 64-fold by age 78. Resilience and biological aging can be improved with lifestyle. In a randomized trial of healthy males, an 8-week diet and lifestyle intervention reversed biological age by 3.2 years (11). Hemodynamic load and inflammation appear to mediate most of the adaptive cardiac changes in HFO. Thus, patients achieving over 20% weight reduction before age 60–70 years may see complete reversal of HF (12).

Patients with a long history of obesity need to be counseled about the cumulative “obesity–years” burden and may benefit from cardiac evaluation. Empagliflozin reduces rehospitalization in HFpEF.^1^ Randomized trial with calorie restriction and exercise derived 7–10% weight loss benefits obese HFpEF patients (13). Gastric bypass studies have shown diastolic cardiac abnormalities are due to obesity and improve when >20% weight loss is achieved (7). The dramatic reductions in symptoms, rehospitalizations, and medication requirements observed in HFO patients who lose 20–35% of body weight may be due intact resilience pathways (14). Based on patient feedback with holistic mind-body methods, we are building metabolic HF clinics targeting several resilience pathways for sustaining weight loss (12). Intentional 15–25% weight loss by 65–70 years in obese symptomatic patients may reverse HFpEF (15).

## Conclusions

HF of obesity manifests at younger age, with fewer associated comorbidities and carries a better prognosis than other HFpEF phenotypes. Masked clinical signs, symptoms and imaging limitations delay diagnosis. The cardiac changes due to severe obesity are predominantly “adaptive.” Resilience in younger HFO may allow regression of cardiac abnormalities with 20–35% weight loss. Based on accumulating evidence, HFO is less a HFpEF phenotype and more a reversible cardiomyopathy with substantial clinical and health policy implications.

## Author Contributions

The author confirms being the sole contributor of this work and has approved it for publication.

## Conflict of Interest

Author AC was employed as an Advisor by the Cardiac Wellness Institute, Chennai, India. AC is co-founder of www.HiLifeJourney.org, a non-profit online self-help tool aimed at improving cardiac health and resilience through self-inquiry. AC is the author of Seeking HUNGER, a book encouraging self-inquiry for holistic health. The author declares that the research was conducted in the absence of any commercial or financial relationships that could be construed as a potential conflict of interest.

## Publisher's Note

All claims expressed in this article are solely those of the authors and do not necessarily represent those of their affiliated organizations, or those of the publisher, the editors and the reviewers. Any product that may be evaluated in this article, or claim that may be made by its manufacturer, is not guaranteed or endorsed by the publisher.
